# Opacification of lenses cultured in the presence of Pb

**Published:** 2010-10-26

**Authors:** R.E. Neal, C. Lin, R. Isom, K. Vaishnav, J.S. Zigler

**Affiliations:** 1 Department of Environmental and Occupational Health Sciences, School of Public Health and Information Sciences, University of Louisville, Louisville, KY; 2 National Eye Institute, National Institutes of Health, Bethesda, MD

## Abstract

**Purpose:**

Occupational and environmental Pb-exposure is associated with protein aggregation diseases which typically present in elderly populations (Parkinsons and cataract). Post-translational processing of crystallins, the major structural proteins of the lens, is altered with short-term Pb-exposure in Fisher 344 rats. In addition, lenses from aged rats become opaque upon long-term exposure to Pb in organ culture. To explore the route to lens opacification in the presence of Pb, cultured lenses from young rats which exhibit higher metabolic activity in lens culture and are more susceptible to experimental cataract in vivo and in vitro were exposed to Pb and evaluated for morphological and biochemical alterations.

**Methods:**

Following culture in Pb (as lead nitrate) for four days (in the presence/absence of oxidative challenge), lenses were examined for clarity, integrity of epithelial layer, and molecular stability including crystallin post-translational modification and choline transport. Clarity of lenses cultured with/without Pb for up to 8 days was assessed to determine if Pb exposure would accelerate opacification.

**Results:**

Lenses cultured in Pb for four days exhibited epithelial abnormalities including epithelial cell multilayering and nuclei abnormalities with extension of the nucleated epithelial cells past the bow region. Alterations in crystallin post-translational modifications and decreased membrane transport of choline were noted without corresponding lens opacification or altered α-crystallin chaperone activity. Lenses treated with Pb according to the same exposure protocol with subsequent challenge by hydrogen peroxide became opaque while the contralateral control lenses did not. Lenses which were cultured in the presence of Pb for longer periods with no subsequent oxidative insult exhibited lens failure at earlier time points than did the controls.

**Conclusions:**

These data indicate that Pb-exposure can accelerate the degradation of the cultured lens through induction of epithelial cell abnormalities, induce structural protein modifications before opacity, and predispose the lens to opacification with subsequent oxidant challenge.

## Introduction

The banning of Pb, a potent environmental toxin, as a pigment, pesticide, and fuel additive has led to decreases in the Pb burden of the general population and of children specifically [1-3]. However, approximately 800,000 children in the United States have blood Pb levels exceeding the Center for Disease Control recommended level of 10 µg/dl or less [4]. Long-term deficits in neurologic, reproductive, and visual function have been associated with in utero, childhood and occupational exposure to Pb [5-11]. Lenticular opacities are associated with decades-prior adult Pb-exposure which supports the hypothesis of Pb as a predisposing effector leading to subsequent lens opacity [12].

The mechanisms related to lenticular opacity associated with Pb-exposure have been explored. Pb accumulation in normal healthy lenses during organ culture or in vivo after oral dosing may be directly related to Pb binding to the lens capsule [13,14]. Pb has a potent effect on glutathione metabolism, metabolic activity, and metal homeostasis in lenses from adult rats exposed for longer periods to low levels of Pb [15,16]. Antioxidant or chelator administration to rats following Pb-exposure can partially alleviate the loss of glutathione and the resultant increases in protein bound thiols [17,18]. Our research has previously established that short-term oral Pb-exposure in 3 month old rats following 5 weeks of Pb-exposure results in altered post-translational modification of the major lens structural proteins, crystallins, without opacification. Additionally, lenses from older rats (4.5 months) became opaque when cultured for three weeks with Pb [17] even though metabolic activity in aged lenses is a fraction of younger lenses. Thus, in vivo and in vitro rodent models of Pb-exposure have implicated oxidative stress and membrane binding as possible mechanisms for lenticular opacification.

With the association of Pb exposure and decades later cataract formation, it seems likely that Pb exposure acts in conjunction with later systemic stressors to induce opacification. The current study examines whether Pb acts as a predisposing effector for lens opacity in conjunction with a secondary oxidative challenge in cultured lenses from young rats (approximately 4–6 weeks of age). Additionally, this study explores the effect of Pb exposure on epithelial transport/cellular structure and crystallin protein homeostasis as possible mechanisms through which Pb may induce opacity.

## Methods

### Materials

Pb nitrate, sodium nitrate, urea, dithiothreitol (DTT), depleted α-lactalbumin, 3-[(3-Cholamidopropyl)dimethylammonio]-1-propanesulfonate (CHAPS), trichloroacetic acid, trifluoroacetic acid, α-cyano-4-hydroxycinnamic acid and sequencing grade trypsin were purchased from Sigma Chemical Company (St. Louis, MO). Immobilized pH gradient (IPG) strips were purchased from BioRad (Hercules, CA). Bis-Tris gels, MOPS running buffer, SilverQuest and Colloidal Blue staining kits were purchased from Invitrogen (Carlsbad, CA).

### Lens culture

Eyes were obtained from Sprague-Dawley (Charles River, Germantown, MD) rats (4–6 weeks of age) immediately after euthanasia following the NIH Animal Research Advisory Committee guidelines. The competent lenses were cultured in modified TC-199 media as previously reported [19,20]. The osmolarity of culture media for all groups was adjusted to 298±2 mOsm. Briefly, lenses were placed in 2.0 ml of modified TC-199 medium in 24 well plates and allowed to equilibrate in an incubator (37 °C, 5% CO_2_) for 2 h; lenses that were damaged during dissection were identified and discarded during this period by measuring protein leakage into the medium. After the equilibration period, surviving “competent” lenses were cultured with 1 µM Pb(NO_3_)_2_ (~20 μg/dl) or unmodified media. The media was changed every other day for a period of 3–9 days. An additional group, 2 µM NaNO_3_ (n=3), was initially included as a counter ion control exposure grouping to assess the impact of nitrate concentration on lens competence. As TC-199 media contains ferric nitrate, the additional nitrate added through addition of the Pb salt doubled the media nitrate content. The lenses that were cultured in NaNO_3_ did not vary from the lenses cultured in unmodified media in hydrogen peroxide clearance, choline uptake, protein disulfide formation or in morphology. The data from the NaNO_3_ (n=3) and unmodified media (n=6) for these assays were combined and represented as the Control group. Subsequent analyses, including the 2D gels and α-crystallin chaperoning assay, used lenses cultured in unmodified media as controls and without the inclusion of a NaNO_3_ group since the added nitrate salts did not affect lens competence.

### Measurement of hydrogen peroxide concentration

For the hydrogen peroxide challenge of lenses cultured in Pb(NO_3_)_2_ (4 lenses per group; Control, 1 µM Pb), the Pb containing media was replaced after 3 days with modified TC-199 supplemented with 250 µM hydrogen peroxide and changed daily. One hundred microliter aliquots of the medium were removed to assay for hydrogen peroxide clearance. H_2_O_2_ concentration was measured after 2 h using a Model 2700 Biochemistry Analyzer (Yellow Springs Instrument Co., Yellow Springs, OH). After 2 days of hydrogen peroxide exposure the lenses were collected and photographed to document alterations in lens clarity. The experiment was repeated 3 times for a total of 12 lenses per group.

### Tritiated choline uptake assay

Tracer levels (0.2 µCi per well) of [^3^H]choline were added to cultures after 4 days in the presence or absence of Pb nitrate (1 µM Pb, 5 lenses per group). After 4 h, the lenses were removed from culture, rinsed with PBS, blotted, and weighed. The lenses were homogenized in 1 ml of cold 10% trichloroacetic acid and the soluble fraction was collected. One hundred microliters of the lens supernatant and 100 ul of the media were counted on a scintillation counter. The lens water (L) to medium (M) concentration ratios (L/M) were calculated as previously published [20]. In brief, the lens weight was multiplied by 0.65 to give the lens water volume. The total counts were adjusted for lens water volume and divided by the total counts per media volume to give the L/M ratio. The experiment was repeated once for a total of 10 lenses per group.

### Protein bound thiols

Following 4 days of culture in the presence or absence of various concentrations of Pb nitrate, lenses were collected and homogenized in 500 µl cold 10% TCA. The protein pellets were washed three times with 10% TCA and then resuspended in 10 mM Tris pH 7.4. One hundred microliters of resuspended pellet was mixed with 10 µl of 2.5% sodium borohydride in 0.01 N NaOH. The mixture was incubated for 1 h at 37 °C. Ten percent TCA was added to a final concentration of 1% to stop the liberation of thiols. The freed thiol levels (nmol/mg) were measured by the Ellman’s assay as previously described [19]. A total of 6 lenses per group were processed and assayed.

### Lens epithelial histology

Three lenses per group were cultured for 3 days in the presence or absence of Pb nitrate as above, fixed in 2.5% glutaraldehyde in cacodylate buffer for 4 h, and transferred to 10% buffered formalin. The fixed samples were embedded in methylmethacrylate, sectioned and stained with hematoxylin and eosin (H&E). The experiment was repeated twice for a total of 9 lenses per group. Three sections per lens were examined for histological alterations.

### Purification of α-crystallin

Three to four lenses per group (control and 1 µM Pb nitrate) were homogenized in 0.5 M Tris buffer (pH 7.4) with 0.1 M KCl, 1 mM EDTA, 10 mM 2-mercaptoethanol, and 0.2% NaN_3_. Following centrifugation at 10,000× g for 30 min at 4 °C, the sample was loaded on a Superose-12 column and eluted with the same buffer with 1 ml fractions collected. The α-crystallin peaks were identified by SDS–PAGE and pooled for reinjection on the same HPLC system [21]. The final α-crystallin fractions were pooled, desalted, lyophilized and stored at −70 °C until use. A total of 24 lenses per group were processed for a resulting yield of 6–8 samples per group (Control and 1 µM PbNO_3_).

### 2D gel electrophoresis

The first dimension isoelectric focusing was performed using a BioRad Protean IEF Cell with 7 cm 3–10NL immobilized pH gradient strips. Aliquots of the purified α-crystallin or whole lens samples (cytoskeletal analysis) were homogenized on ice with 8 M urea containing 4% CHAPS and 100 mM DTT (rehydration buffer). No insoluble material was visible. Four micrograms of lens protein was loaded onto the IPG strips with rehydration buffer containing 2% ampholytes and bromophenol blue for a total volume of 125 μl. IEF strips were rehydrated for 12 h and then a three step program was used including Step 1: initial removal of salts (500 V for 500 V-h); Step2: an intermediate step (1000 V for 2000 V-h); and Step 3: the final protein separation according to pI (8000 V for 16,000 V-h). Following protein IEF focusing, the IPG strips were equilibrated with 100 mg/10 ml DTT and 400 mg/ml iodoacetamide and the second dimension SDS–PAGE was performed using 12% pre-cast Bis-Tris gels and MOPS as the running buffer. The gels were fixed and stained with either silver or colloidal Coomassie blue G-250. The gels were scanned with a Molecular Dynamics Personal Densitometer and analyzed using Phoretix image analysis software (Nonlinear Dynamics, Newcastle upon Tyne, UK). Six samples from the 1 µM Pb and 6 samples from the control group were analyzed in duplicate.

### Mass spectrometry

Protein spots of interest were cut from the gel, destained in 50% methanol in 20 mM ammonium bicarbonate buffer, and digested overnight with 10 ng/ul trypsin [22]. Peptides were extracted in successive washes of aqueous acetonitrile (50% acetonitrile) and acetonitrile containing 0.1% trifluoroacetic acid (TFA) and plated on a steel target (α-cyano-4-hydroxycinnamic acid as the matrix). Data acquisition was performed on a Voyager DE-STR MALDI-TOF-MS (Applied Biosystems, Carlsbad, CA) in the reflectron mode with 150 ms delay time and an average of 100 spectra collected. Protein identification was based on 4 or more dominant masses corresponding to unmodified peptides from a protein with a peptide mass deviation of less than 0.03% which gave the highest MOWSE score [23] upon searching of the NCBInr.2005.06.01 database by Protein Prospector algorithm.

### Measurement of α-crystallin chaperone activity

α-Crystallin and apo-α-lactalbumin (1:3 weight ratio) were dissolved in 50 mM phosphate buffer (pH 6.82) containing 0.1 M NaCl and 2 mM EDTA. DTT at a final concentration of 50 mM was added to initiate denaturation of the lactalbumin. Aggregation was monitored as a function of lactalbumin aggregation as previously described [21].

### Statistical analysis

Student’s *t*-tests were calculated (GraphPad Prism 4.0; GraphPad Software Inc., La Jolla, CA) to determine statistical significance of the mean ±standard deviation of the Pb groups versus the control groups for all assays.

## Results

### Lenses cultured in Pb fail at an accelerated rate compared with control lenses

**Figure 1:**
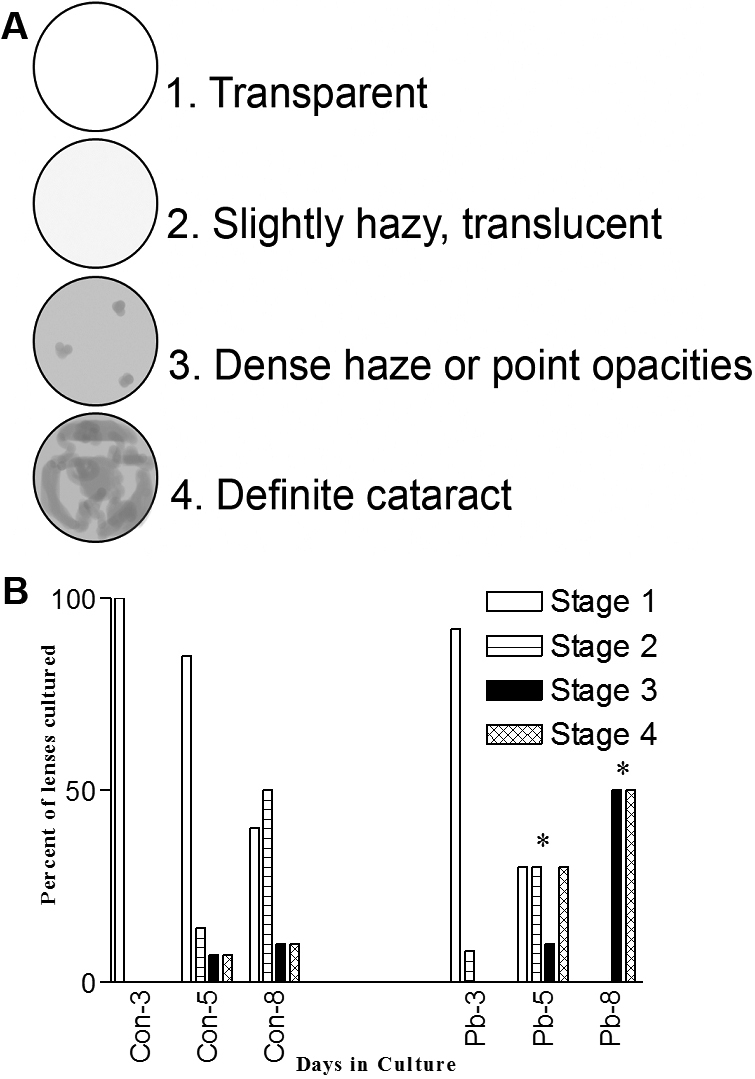
Pb accelerates opacification of cultured lenses. **A**: Lens opacity grading standards. **B**: Lenses cultured in Pb containing media develop opacities at a faster rate than control cultured lenses.

To examine whether longer term culture of young lenses in the presence of Pb would lead to lens opacity formation (as does long-term culture of older lenses) without subsequent oxidative challenge, lenses were cultured up to 9 days in the presence or absence of Pb nitrate. The lens clarity was monitored and graded daily according to the chart shown in Figure 1A. Lenses cultured for 3 days in the presence or absence of Pb were transparent (Figure 1B). The clear lenses were weighed and the degree of radiolabeled sulfur-containing amino acid incorporation was measured with no differences observed between the control and Pb (data not shown). This indicates that short-term Pb-exposure does not induce osmotic swelling or lens shrinkage in lens organ culture and the lenses remain competent to synthesize new protein. This was expected due to the previously reported prolonged culture times necessary for opacification of older lenses in the presence of Pb [17] and the lack of cataract formation following oral Pb dosing in young rodent models.

For control lenses, at culture day 5, roughly 80% of the lenses were at Stage 1, 10% at Stage 2, and 5% at Stages 3 and 4. By culture day 8, 50% of the lenses had progressed to Stage 2, with approximately 7% at Stages 3 and 4. This indicates that 8 days in culture is approaching the outer limits of lens viability under the conditions employed. In contrast, by day 5 in culture with Pb only 30% of the lenses remained at Stage 1. At culture day 8, the Pb exposed lenses have all advanced to Stage 3 and 4 indicating that lenses cultured in the presence of Pb (without subsequent secondary oxidative challenge) fail at an accelerated rate as compared to the control lenses.

### Hydrogen peroxide challenge induces lenticular opacities in lenses cultured with Pb

**Figure 2:**
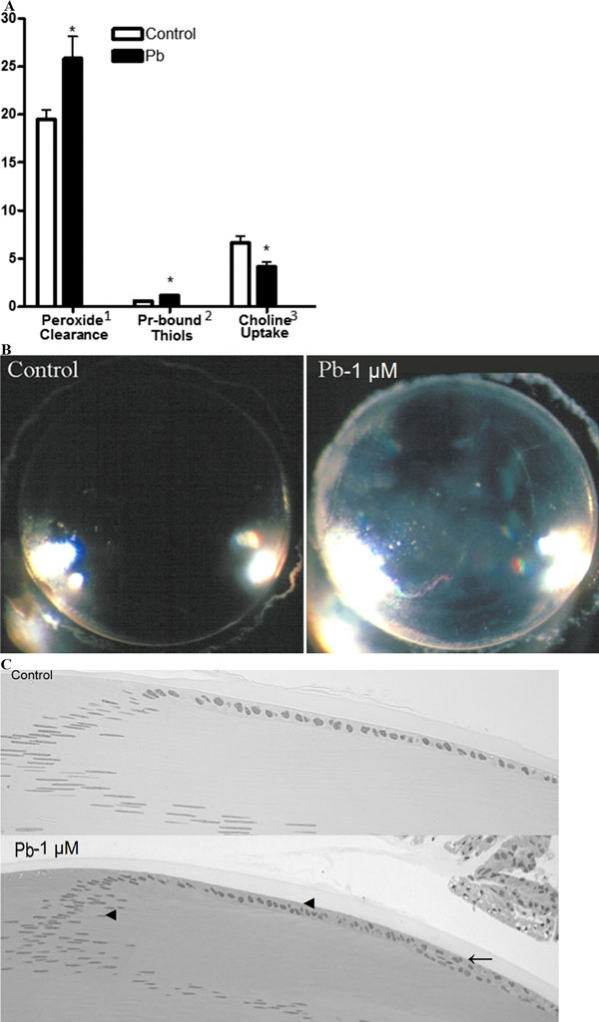
Pb predisposes lenses to opacification in culture.** A**: Increased clearance of H_2_O_2_ (250 µM) from the culture media, increased protein bound thiols (Pr-bound thiols), and decreased choline uptake for lenses cultured with Pb (3 days) as compared to the control lenses. **B**: Lenses cultured with 250 µM H_2_O_2_ following culture in the presence of Pb (3 days; 1 uM Pb(NO_3_)_2_) induced lens opacities in the Pb exposed lenses but not the control lenses. **C**: Histology of the bow region of Pb-cultured lenses (3 days without peroxide challenge; 1 uM Pb(NO_3_)_2_), magnification 20× for H&E sections. Epithelial cell doubling, irregular nuclei, and nuclei loss were evident in the Pb exposed samples (as shown by arrowheads).

To examine whether lenses cultured in the presence of Pb are predisposed to failure, lenses were cultured for 3 days in the presence or absence of Pb nitrate and then challenged for 2 days with 250 µM hydrogen peroxide. Lenses cultured with Pb exhibit a higher clearance of hydrogen peroxide from the media than the control lenses (Figure 2A) at 2 h after initiation of oxidative challenge. This may be due to an increase in catalase activity similar to that which has previously been reported for red blood cells collected from Pb exposed animals. Control lenses challenged with 250 µM hydrogen peroxide for 2 days remain clear (5 days total culture) but the Pb cultured lenses could not withstand this oxidative challenge and developed slight to moderate opacities located in the subcapsular space extending from the capsule boundary to cortical region in most instances (Figure 2B) with rare involvement of the nuclear regions. Complete lens opacification was noted by the beginning of day 7 for all of the Pb-exposed lenses with ~80% of control lenses remaining clear (stages 1 and 2 of grading scale in Figure 1A).

### Lens membrane transport decreased and protein oxidation increased following culture with Pb

The degree of protein mixed disulfides present, a measure of protein oxidation and oxidative stress, was increased in Pb-exposed lenses (4 days; Figure 2A). In Figure 2A, the effect of Pb on the active transport of tritiated choline, a sensitive indicator of lens damage [24], from the culture medium is also shown. Statistically significant decreases in the level of tritiated choline accumulated by the Pb exposed lenses are evident as compared to the controls. Together, the alterations in epithelial nutrient transport and markers of increased oxidative stress may predispose the lens to failure with a subsequent challenge.

### Epithelial histology is altered after Pb culture

Alterations in the lens epithelium following culture in 1 μM Pb for 3 days as compared to the control cultured lenses (Figure 2C) include smaller and irregularly shaped nuclei anterior to the bow region and multilayering of cell layer. The controls exhibit a single cuboidal epithelial layer with regularly spaced cells with ovoid nuclei. No differences were evident in the posterior fiber cell region of the lenses (data not shown). These data imply that culture in Pb containing media induces abnormalities in epithelial cells including defects in membrane transport and proliferation.

### α-crystallin 2D gel patterns are altered following lens culture with Pb nitrate

**Figure 3:**
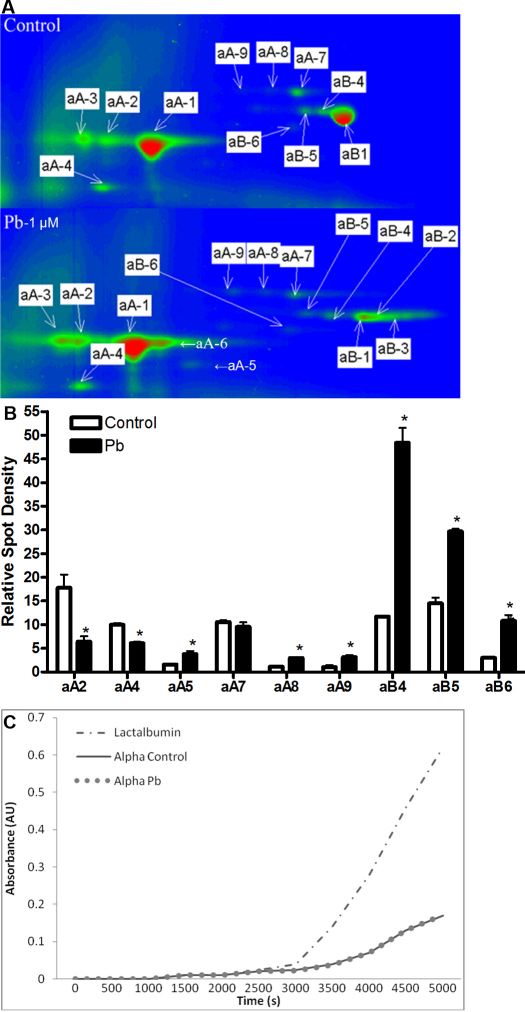
Pb alters α-crystallin processing in cultured lenses. **A**: Two dimensional gels of HPLC purified α-crystallin from cultured lenses (3 days; 1 uM Pb(NO_3_)_2_). **B**: Quantification of the relative amounts of various post-translationally modified forms of α-crystallin in cultured lenses (3 days; 1 uM Pb(NO_3_)_2_). Spot densities (normalized to either dominant αA- or αB-crystallin protein spot) of different isoforms of α-crystallin are altered with Pb-exposure (3 days; 1 uM Pb(NO_3_)_2_). Only protein spots sufficiently resolved from neighboring protein spots were quantified. **C**: α-Crystallin purified from lenses exposed in vitro to Pb (3 days; 1 uM Pb(NO_3_)_2_) does not exhibit alterations in chaperone function. Dash/dot line (- - -) shows aggregation of denatured lactalbumin without α-crystallin. Solid line (—) shows aggregation of denatured lactalbumin in the presence of α-crystallin purified from lenses cultured in control media. Unconnected circles (o o o) show aggregation of denatured lactalbumin in the presence of α-crystallin purified from lenses cultured in the presence of Pb.

α-Crystallin, a molecular chaperone and major lens structural protein, is believed to maintain lens transparency through prevention of protein aggregation. Post-translational modification of α-crystallin may negatively affect maintenance of lens clarity through decreased chaperone function. To examine the degree of post-translational modification of α-crystallin in Pb exposed lenses, α-crystallin was purified from lenses cultured for 4 days in the presence or absence of Pb and separated by two dimensional gel electrophoresis with the individual protein spot intensities quantified by densitometry. A comparison of the 2D gel spot patterns obtained from 10µg of protein from control and Pb exposed lenses is shown in Figure 3A. The dominant αA-crystallin form is denoted as αA1-crystallin with an apparent molecular weight of 22 kDa and pI of 6.3 in both the control and Pb exposed lenses. Several αA-crystallin spots which are related to αA1-crystallin are present in the control and Pb exposed lenses representing both modified (αA2- and αA3-crystallin) and cleaved (αA4-crystallin) forms of this same αA-crystallin isoform. The apparent molecular weight of both αA2- and αA3-crystallin is 22 kDa while αA4-crystallin is 17 kDa. These αA-crystallin spots are more acidic than the dominant αA1-crystallin spot, ranging from a pI of 5.7 to 6.0. Spot αA5-crystallin is barely visible on the gel from control cultured lenses but is clearly visible on the gels from lenses cultured in the presence of Pb. Spot αA5-crystallin has an apparent molecular weight of 19 kDa and a pI of 6.45. Three additional spots (αA7–9-crystallin) were identified as an alternatively spliced form of αA-crystallin, the αA_ins_-crystallin form, which is present in rodents but not humans or other primates [25].

Six forms of αB-crystallin are visible on the gels shown in Figure 3A. Spots αB1-, αB4-, αB5-, and αB6-crystallin are present on gels from both the control and Pb exposed lenses. Spots αB2- and αB3-crystallin, present only in the Pb-exposed lenses, are more basic but the same molecular weight as the dominant αB-crystallin spot, αB1-crystallin. The spot boundary of the αB2- and αB3-crystallin spots varies from gel to gel which may be indicative of poor separation in the basic region of the first dimension IEF focusing. Spots αB1- through αB5-crystallin have the same apparent molecular weight of 24 kDa and range in pI from 6.8 to 7.4. Spot αB6-crystallin has a molecular weight of 23 kDa and pI of 6.7.

Quantitation of the relative spot densities of the different forms of α-crystallin is shown in Figure 3B (only protein spots sufficiently resolved for quantitation and whose abundances are altered are reported). For the αA-crystallin spots, a relative decrease in more acidic forms of αA-crystallin, both cleaved and modified forms (Spots αA2- and αA4-crystallin), was observed in the lenses cultured in the presence of Pb as compared to the control lenses. The relative abundance of the more basic cleaved form, αA5-crystallin, is increased in the Pb exposed lenses. For the αA_ins_-crystallin isoform, spots αA8- and αA9-crystallin are increased in abundance in the Pb cultured lenses when normalized against the dominant αA1-crystallin spot. The spot αA7-crystallin does not change significantly in the Pb cultured lenses as compared to the control cultured lenses. This indicates alterations in the normal processing of αA-crystallin do occur in optically transparent lenses which have been cultured in the presence of Pb nitrate. It is also interesting that the total amount of αA_ins_-crystallin appears to increase in lenses cultured with Pb nitrate since there is approximately 5%–10% more of two of the three spots identified as αA_ins_-crystallin for a total change in the amount of αA_ins_-crystallin by 10%–20%. This result was not statistically significant but the trend was evident in nine out of twelve individual lenses examined in the Pb exposed group.

Three forms of αB-crystallin which are present in both the control and Pb cultured lenses, representing more acidic (αB4- and αB5-crystallin) and cleaved forms (αB6-crystallin), have significant increases in relative abundance in the Pb cultured lenses when normalized against the dominant spot form of αB-crystallin (αB1-crystallin; Figure 3B). Spots αB2- and αB3-crystallin are only found on gels from the Pb exposed lenses and represent basic modifications of αB1-crystallin which could not be sufficiently resolved to quantitate αB1-crystallin on a small format gel.

### α-Crystallin chaperone activity unaltered by lens culture in the presence of Pb for 4 days

The degree of aggregation of chemically denatured lactalbumin is decreased in the presence of α-crystallin (Figure 3C). The lactalbumin without α-crystallin trace (dotted/dashed line), shows an absorbance intensity of 0.55 at 4,500 s. When lactalbumin is denatured in the presence of α-crystallin purified from lenses cultured with or without Pb nitrate, no differences in the absorbance spectra from 0 to 4,500 s are observed which demonstrates an equivalent α-crystallin chaperone activity. Short-term lens culture in the presence of Pb, which alters the post-translational modification profile of α-crystallin, does not alter chaperoning activity.

## Discussion

Pb is an ocular toxin which negatively affects the lens antioxidant balance and accumulates in opaque human lenses [15,26]. Epidemiological evidence identifying elevated tibial Pb levels as a risk factor for cataract decades after the initial Pb-exposure supports the hypothesis that Pb-exposure may be associated with impaired lens competence [12]. Whether Pb acts as direct lenticular toxin or is an associative marker for causal agents that may include poor diet, increased risk of diabetes, or other environmental exposures remains controversial [27]. The current in vitro study examined the ability of Pb to predispose the lenses of young rats (4–6 weeks old) to opacification or to directly induce lenticular abnormalities. The present study employed a Pb level which is physiologically relevant (1 µM, 20 µg/dl) although the levels of Pb in ocular fluids after chronic or acute in vivo Pb-exposure at this dosage are unknown. This Pb level was sufficient to induce lens opacification, though at the outer time limit of which rodent lenses can be organ cultured. This may support the hypothesis that Pb is a slow-acting ocular toxin with long-term exposure increasing the risk of ocular pathology.

The mammalian lens is composed of a single layer of nucleated cuboidal epithelial cells which differentiate at the bow region into elongated lens fiber cells which at maturity lack nuclei and organelles [28]. Rat lenses cultured in the presence of Pb exhibited abnormalities in the lens epithelial cell layer after four days. Of particular interest are the cell layer multilayering and abnormal morphology of nuclei, which coupled with the decrease in choline uptake, indicate significant alterations in membrane integrity and function and possibly in cell proliferation. Carrier assisted choline transport into healthy lenses primarily supports phosphocholine synthesis, membrane generation, and cell synthesis with deficits in choline uptake preceding cataractogenesis. The region where epithelial cells differentiate into mature fiber cells also exhibited abnormalities including the extension of nucleated cells past the bow region and the presence of vacuoles in a few of the lenses exposed to Pb (data not shown). These studies demonstrate that Pb can negatively impact lens epithelial cell proliferation. Examination of the impact of Pb-exposure on the retina, following either developmental or adult exposure, identified decreases in the number of rods and bipolar cells and in rhodopsin levels which persisted after the initial Pb insult [29]. Together, these studies demonstrate that physiologically relevant dosages of Pb can negatively impact ocular tissue structure. The effect of Pb exposure on the vitreo-retinal interface and the vitreous humor physico-chemical homeostasis including the oxygen diffusion pattern as well as the transport of Pb into aqueous and vitreous humor is of considerable interest for future studies.

Crystallins, the dominant structural proteins of the lens which account for over 90% of the total protein complement, are responsible for lens transparency and refractivity. Post-translational modifications of α-, β-, and γ-crystallins occur during normal lens development, however alterations in protein processing have also been associated with cataract formation [22,30-35]. The two isoforms of α-crystallin (αA- and αB-crystallin) share approximately 60% sequence homology. αB-crystallin is found in non-ocular tissues and can be upregulated in response to various tissue stresses [36-40].

The current study examined whether alterations in the post-translational modification of the two α-crystallin isoforms occurred in the whole lens in conjunction with the epithelial abnormalities induced by Pb-exposure. The observed increase in protein mixed disulfides with increased abundance of acidic and cleaved forms of αA- and αB-crystallin before lens failure indicates alterations in oxidative post-translational modification of lens proteins does occur before lens opacification. Similar trends in alterations of αA-crystallin post-translational modifications were observed in vivo following short-term oral Pb-exposure where the lenses remained transparent [17]. However, the relative abundance of modified αB-crystallin isoforms was not increased in the in vivo model. This may be due to the inability to differentiate these modified αB-crystallin isoforms from other crystallins such as β- and/or γ-crystallin in the prior study of whole lens crystallin proteins. Conversely, the data presented in this study may point to a general hierarchy of modification of the two α-crystallin isoforms (αA- and αB-crystallin) which is weighted toward early response to chronic toxicant challenge by αA-crystallin followed later by αB-crystallin modification. Due to the variance in effective dosage and time course between the in vitro and in vivo model, this question is not answered by comparison between the two studies. The present study supports the hypothesis that Pb-exposure induces alterations in crystallin processing before opacification–indicating that not only is membrane transport and morphology aberrant following short-term culture with Pb, but compromised protein processing resulting in altered thiolation, cleavage, and non-specified modifications which result in isoelectric point shifts for the soluble dominant lenticular proteins occurs.

The question of whether protein functional alterations were evident before opacification was also addressed. Alpha-crystallin has been identified as a molecular chaperone of the sHsp family which prevents protein aggregation due to cellular stresses including oxidative damage [41]. α-Crystallin purified from lenses exposed to Pb for 4 days did not exhibit decreased chaperone function as compared to α-crystallin purified from the control lenses, even though the post-translational processing of α-crystallin was altered at this time point. We did not assess the impact of longer term culture in the presence of Pb on chaperone function as significantly greater proportions of lenses were progressing toward complete lens failure by day 5 of culture in this group with insoluble protein aggregates visible. Though the data are not presented here, stage 3 and 4 lenses (~50% of lenses on day 5 in the Pb-group) exhibited dramatic cleavage of all crystallins present on 2D gels and swelling of the lenses was evident.

This indicates that the observed opacification following the addition of a 250 μM hydrogen peroxide challenge for the Pb-exposed lenses, but not controls (much higher concentrations of peroxide are needed to induce opacities in normal cultured lenses [42]), is most likely due to the observed membrane degradation coupled to oxidation-induced protein aggregation. This could be due to increased hydrogen peroxide penetration of the membrane or decreased antioxidant reservoirs in the Pb-exposed lenses. Our prior study of the effects of oral Pb-exposure on rodent lenses found that the lenticular reduced glutathione pool is compromised [18,43]. Though we have demonstrated that Pb-exposure alone (4 days culture) does not affect α-crystallin chaperoning ability—it is highly likely that thiolation of α-crystallin has occurred with Pb-exposure since increased protein thiolation was observed. For future studies, it may be interesting to examine whether thiolation alters α-crystallin chaperoning capacity following a subsequent oxidative challenge. If so, the opacification of the Pb-exposed lenses observed following hydrogen peroxide exposure could be partially related to inadequate chaperone availability due to a specific post-translational modification. The data presented in the current paper does not address this point but does support Pb as a toxin which predisposes the lens to failure with clear alterations in the epithelial cell layer before secondary oxidant challenge and opacification.

The current study also examined whether lenses from young rats (4–6 weeks) cultured in the presence of Pb for longer periods of time would develop opacities. The significantly increased rate of failure of Pb cultured lenses was associated with development of lenticular opacities, severe abnormalities of the epithelial layer, increased weights, and pronounced acidification and degradation of crystallins (data not shown). Lenses cultured in unmodified media exhibited the same types of abnormalities upon failure, though failure was at a later time point. Thus Pb may accelerate the decline in lens membrane function ultimately leading to cataract formation in both young lenses and in the older, less metabolically active lenses (from 4.5 month old rats) from our prior study [17]. We propose that Pb induces oxidative imbalances in clear lenses which lead to the epithelial abnormalities and later to opacification with/without additional oxidative challenge. Further, we propose that these oxidative imbalances precede the epithelial membrane degradation since no evidence of such damage was found in our in vivo studies though oxidative stress was evident (unpublished data).

This study has demonstrated the ability of Pb to compromise lens integrity following short-term in vitro challenge and to predispose the lenses from 4 to 6 week old rats to opacities induced by subsequent oxidative challenge. Pb-exposure also negatively impacted lens clarity at longer exposure times which may point to a direct cataractogenic effect of Pb. Coupled with our previous in vivo data demonstrating alterations in lens homeostasis following short-term oral Pb-exposure in young rats, these data support the hypothesis that Pb-exposure is causally related to lens opacification. While there is a lack of knowledge of the type of cataract associated with Pb-exposure in humans, the current study raises the question of whether cortical opacities and lens epithelial cell biochemical abnormalities may be linked to in vivo Pb-exposure in humans.
